# Nodal Global Efficiency in Front-Parietal Lobe Mediated Periventricular White Matter Hyperintensity (PWMH)-Related Cognitive Impairment

**DOI:** 10.3389/fnagi.2019.00347

**Published:** 2019-12-10

**Authors:** Haifeng Chen, Lili Huang, Dan Yang, Qing Ye, Mengdi Guo, Ruomeng Qin, Caimei Luo, Mengchun Li, Lei Ye, Bing Zhang, Yun Xu

**Affiliations:** ^1^Department of Neurology, Drum Tower Hospital, Medical School and The State Key Laboratory of Pharmaceutical Biotechnology, Institute of Brain Science, Nanjing University, Nanjing, China; ^2^Jiangsu Key Laboratory of Molecular Medicine, Medical School of Nanjing University, Nanjing, China; ^3^Jiangsu Province Stroke Center for Diagnosis and Therapy, Nanjing, China; ^4^Nanjing Neuropsychiatry Clinic Medical Center, Nanjing, China; ^5^Department of Radiology, Affiliated Drum Tower Hospital of Nanjing University Medical School, Nanjing, China

**Keywords:** cognitive impairment, cingulo-opercular network, functional efficiency, fronto-parietal network, white matter hyperintensity

## Abstract

White matter hyperintensity (WMH) is widely observed in the elderly population and serves as a key indicator of cognitive impairment (CI). However, the underlying mechanism remains to be elucidated. Herein, we investigated the topological patterns of resting state functional networks in WMH subjects and the relationship between the topological measures and CI. A graph theory-based analysis was employed in the resting-state functional magnetic resonance scans of 112 subjects (38 WMH subjects with cognitive impairment without dementia (CIND), 36 WMH subjects with normal cognition and 38 healthy controls (HCs), and we found that WMH-CIND subjects displayed decreased global efficiency at the levels of the whole brain, specific subnetworks [fronto-parietal network (FPN) and cingulo-opercular network (CON)] and certain nodes located in the FPN and CON, as well as decreased local efficiency in subnetworks. Our results demonstrated that nodal global efficiency in frontal and parietal regions mediated the impairment of information processing speed related to periventricular WMH (PWMH). Additionally, we performed support vector machine (SVM) analysis and found that altered functional efficiency can identify WMH-CIND subjects with high accuracy, sensitivity and specificity. These findings suggest impaired functional networks in WMH-CIND individuals and that decreased functional efficiency may be a feature of CI in WMH subjects.

## Introduction

Cerebral small vessel disease (CSVD) is a group of clinical syndromes arising from impaired blood circulation in cerebral arterioles, small perforating arteries, capillaries and small veins in the brain. CSVD accounts for approximately 10–30% of ischaemic strokes worldwide (Patel and Markus, 2011; Xu et al., 2018) and is a leading vascular contributor to cognitive deficits and dementia, mainly involving global cognitive function, information processing speed, and executive function (Jokinen et al., 2009; Debette and Markus, 2010; Wu et al., 2016; Liu et al., 2019). The early prediction of cognitive impairment (CI) due to CSVD is of great value because managing vascular risk factors early, e.g., diabetes, hypertension, hyperlipidaemia and smoking, may decrease the risk of developing CI in individuals with CSVD (Gorelick et al., 2011; Gu et al., 2019).

White matter hyperintensities (WMH), subcortical/lacunar infarcts, an enlarged perivascular space and brain microbleeds have been perceived as radiological manifestations of CSVD (Ter Telgte et al., 2018). Many studies have investigated the association between these image representations and CI in CSVD subjects, and conflicting results have been shown. WMH is thought to be detected in 72–96% of the population over 60 years old (De Leeuw et al., 2001; Zhuang et al., 2018; Lampe et al., 2019). Increasing evidences have confirmed that WMH burden can lead to CI in executive function and information processing speed and associated with increased risk of dementia (Van Dijk et al., 2008; Sudo et al., 2013). However, WMH is very common in elderly subjects with normal cognition, and a portion of these individuals do not develop CI (Lampe et al., 2019). Similarly, although a link between subcortical/lacunar infarcts and CI has been shown by neuropathological studies (Raman et al., 2014), subcortical/lacunar infarcts are also commonly observed in elderly subjects without CI or with non-vascular CI (Brundel et al., 2012). The association between brain microbleeds and CI varies according to the counts and location of the lesions (Banerjee et al., 2016), but some studies have reported no association (Brundel et al., 2014; Heringa et al., 2014). Finally, the association between enlarged perivascular space and cognition in CSVD is also conflicting (Hurford et al., 2014; Yao et al., 2014). These findings suggest that the structural lesions of CSVD are not always associated with CI. Exploring the functional mechanisms underlying CI in CSVD would contribute to the understanding of the onset of CI in CSVD.

In the last two decades, a graph theory-based analysis method has been developed to explore brain functional connectivity (FC) networks in both resting or task states, and these networks can be constructed with functional neuroimaging data retrieved from fMRI blood-oxygen-level-dependent (BOLD) time series. Related studies have demonstrated that human brain large-scale functional networks are composed of brain regions highly correlated at the level of neural activity, partly reflecting the underlying structural connectivity architecture confirmed by diffusion tensor imaging scans (Smith et al., 2009; Van Den Heuvel et al., 2009). CSVD exerts its action on connectivity between structural and functional networks, thereby disrupting efficient communication in the brain network (Ter Telgte et al., 2018). A number of studies have investigated structural network connectivity derived from the integrity of white matter tracts in CSVD and the association between alterations in structural network connectivity and CI in CSVD. The network efficiency of the structural network was found to be decreased in CSVD subjects and correlated better with CI than traditional radiological features, e.g., WMH burden (Lawrence et al., 2014; Kim et al., 2015); in addition, lower network efficiency predicted conversion to dementia in CSVD patients (Tuladhar et al., 2016). In contrast, topological patterns of large-scale functional networks have rarely been studied in populations with CSVD. Functional brain alterations have been thought to arise prior to structural changes and clinical symptoms in subjects with non-vascular CI (Sheline and Raichle, 2013). Functional abnormalities can even be detected in healthy subjects at risk for CI (Nichols et al., 2012; Heise et al., 2014). Considering the potential link between functional networks and structural networks, we hypothesized that subjects with WMH, one of the most common images found in CSVD, would display altered topological patterns in large-scale functional networks, and these functional alterations would be associated with CI in subjects with WMH.

White matter hyperintensity subjects with cognitive impairment without dementia (CIND), WMH subjects with normal cognition and healthy subjects were recruited for the present study. All subjects underwent standard neuropsychological tests and multimodal MRI scans. We aimed to investigate topological patterns of resting state functional networks in WMH subjects with and without CI and the relationship between topological measures and CI.

## Materials and Methods

### Participants

The present investigation is a hospital-based cross-sectional study (Clinical Trial: ChiCTR-OOC-17010562) that consists of 112 right-handed participants (74 subjects with moderate-to-severe WMH and 38 matched healthy controls) between 50 and 80 years old. Subjects with moderate-to-severe WMH were divided into WMH-normal cognition (WMH-NC, *n* = 36) and WMH-CI (WMH-CIND *n* = 38) based on the Beijing version of the Montreal Cognitive Assessment (MoCA-BJ). In order to avoid the level of education affecting the results of this scale, the optimal cutoff points are determined according to education level (or years of education). For subjects with no formal education, the MoCA-BJ cutoff was 13/14; for subjects with 1–6 years of education, the MoCA-BJ cutoff was 19/20; and for subjects with seven or more years of education, it was 24/25 (Lu et al., 2011; Yu et al., 2012; Liu et al., 2019). Moderate-to-severe WMH was defined by neuroimaging evidence: Fazekas visual rating scales of grade 2 or 3. Exclusion criteria included cerebral hemorrhage, non-vascular-related WMH mimics (e.g., brain irradiation and multiple sclerosis), dementia (Clinical Dementia Rating Scale ≥1), Mini-Mental State Examination (MMSE) ≤23 and psychiatric disorders. This study was approved by the Ethics Committee of Nanjing Drum Tower Hospital, and each participant provided written informed consent.

### MRI Scanning

All of the participants were examined on a Philips 3.0-T scanner (3.0 T Ingenia (32-channel head coil), Philips, Eindhoven, Netherlands) The examination protocol included a high-resolution T1-weighted turbo gradient echo sequence [repetition time (TR) = 9.8 ms, flip angle (FA) = 8°, echo time (TE) = 4.6 ms, FOV = 250 × 250 mm^2^, number of slices = 192, acquisition matrix = 256 × 256, thickness = 1.0 mm], a FLAIR sequence [TR = 4500 ms, TE = 333 ms, time interval (TI) = 1600 ms, number of slices = 200, voxel size = 0.95 × 0.95 × 0.95 mm^3^, acquisition matrix = 270 × 260] and a gradient-recalled echo planar imaging sequence (TR = 2000 ms, FA = 90°, TE = 30 ms, number of slices = 35, acquisition matrix = 64 × 64, FOV = 240 × 240 mm^2^, thickness = 4 mm). The Wisconsin White Matter Hyperintensities Segmentation Toolbox^1^ was used to automatically determine the total WMH volume, including deep-WMH (DWMH) and periventricular-WMH (PWMH), based on T1-weighted and FLAIR images. The total brain volume was automatically obtained using Statistical Parametric Mapping (SPM8)^2^. Given the skewed distribution of the WMH volumes, the values were first normalized by the corresponding total brain volume and then log-transformed (base 10).

### Neuropsychological Measurement

All subjects underwent a standardized neuropsychological test protocol, including global cognitive assessments and multiple cognitive domain examinations. Global cognitive function was evaluated by MMSE and MoCA-BJ. The raw test scores were converted to *Z*-scores, which were used to calculate the compound cognitive index. Episodic memory was calculated as the mean of the *Z*-scores from the Wechsler Memory Scale-Visual Reproduction-delayed recall and Auditory Verbal Learning Test-delayed recall scores. Visuospatial function is a compound score that includes the mean of the Z-scores of the Clock Drawing Test and Visual Reproduction–copy test. Information processing speed was calculated as the average *Z*-scores of the Trail Making Test-A (TMT-A) and the Stroop Color and Word Tests A and B (Stroop A and B). The language function consisted of the Category Verbal Fluency test and Boston Naming Test. Executive function is a compound score of the average *Z*-scores of the Digit Span Test-backward, TMT-B and Stroop C.

### Image Pre-processing

Resting-state fMRI data were pre-processed by the Graph Theoretical Network Analysis Toolbox^3^. The first ten volumes were discarded before slice time correction and realignment to the first volume to correct for head motion. No participant performed a displacement >2 mm or an angular rotation >2° in any direction. Next, the obtained images were spatially normalized to the Montreal Neurological Institute space and resampled with a resolution of 3 × 3 × 3 mm voxels. Subsequently, filtering was performed using a bandpass filter (0.01–0.1 Hz), and linear trends were also removed. Finally, several nuisance variables, including the Friston-24 parameters and white matter and cerebrospinal fluid signals, were removed by multiple linear regression analysis.

### Network Construction

To define the network nodes, Dosenbach’s atlas was used to parcellate the whole brain into 160 functionally segregated regions of interest (ROIs, radius = 5 mm). These 160 brain regions could be partitioned into six subnetworks: the default mode network (DMN), the fronto-parietal network (FPN), the cingulo-opercular network (CON), the sensorimotor network (SMN), the occipital network (ON), and the cerebellum network (CN). To define the network edge, we calculated Pearson correlation coefficients for each pair of 160 ROIs between the regional mean time series. A Fisher Z-transformation was used to improve the normality of the correlation coefficients. We applied a set of sparsity thresholds (ranging from 0.1 to 0.4, with steps of 0.01) to generate a binary undirected network. This range of sparsity thresholds was chosen because networks were not fully connected at lower sparsity thresholds and were less likely to maintain small-world architecture at higher sparsity thresholds.

### Graph Theory Analysis

We used the Brain Connectivity Toolbox^4^ to calculate two graph theoretical measures including global and local efficiency at the network and node levels, respectively. Functional efficiency measures how efficiently information is exchanged over the distributed brain regions. Human brain networks are seen as systems that are both globally and locally efficient. The detailed descriptions of these metrics are given below.

Global efficiency measures the parallel information transfer ability of the network, which can be computed as follows:

(1) $$E\,g\,l\,o\,b\,a\,l\,\left(G\right)=\frac{1}{N\,\left(N-1\right)}\,\sum_{i\neq j\in G}\frac{1}{d\,i\,j}$$

where *N* is the number of nodes in the network *G*, and *d*_*ij*_ is the shortest path length between node i and j in the network.

Local efficiency measures the information exchanging ability among a sub-graph with locally interconnected nodes; it reflects system redundancy and tolerance to attack, which can be computed as follows:

(2) $$E\,l\,o\,c\,\left(G\right)=\frac{1}{N}\,\sum_{i\in G}E\,g\,l\,o\,b\,a\,l\,\left(G\,i\right)$$

where *N* is the number of nodes in the network *G*, and *G*_*i*_ is the sub-graph consisting of node i and its local neighbors.

To determine the nodal properties of the networks, we computed the nodal global efficiency and nodal local efficiency. The nodal efficiency quantifies the importance of the nodes for information communication within the network.

The nodal global efficiency measures the average shortest path length between a given node *i* and all of the other nodes in the network, which can be computed as follows:

(3) $$E\,n\,o\,d\,a\,l\,\_\,g\,l\,o\,b\,a\,l\,\left(i\right)=\frac{1}{N-1}\,\sum_{i\neq j\in G}\frac{1}{d\,i\,j}$$

where *N* is the number of nodes in the network *G*, and *d*_*ij*_ is the shortest path length between node i and j in the network.

The nodal local efficiency measures the global efficiency of the sub-graph formed by this given node’s neighbors, which can be computed as follows:

(4) $$E\,n\,o\,d\,a\,l\,\_\,l\,o\,c\,\left(i\right)=\sum_{i\in G}E\,g\,l\,o\,b\,a\,l\,\left(G\,i\right)$$

where *G*_*i*_ is the sub-graph consisting of node i and its local neighbors.

Moreover, we also calculated the area under the curve (AUC) for each efficiency metric over the range of sparsity (0.1∼0.4), which provided a summarized scalar for the brain topological characterization independent of single threshold selection.

### Statistical Analysis

Differences across HC, WMH-NC and WMH-CIND groups in demographic and cognitive performance were assessed using chi-squared (χ^2^) tests or one-way analysis of variance (ANOVA) using Statistical Package for Social Sciences (SPSS) version 22 (IBM Corp., Armonk, NY, United States). WMH volumes were compared in SPSS by Kruskal-Wallis one-way ANOVA. The significance level was set at *P* < 0.05.

The AUC of the global and local efficiency over a wide range of thresholds of the network were analyzed with ANOVA to detect significant differences (with Bonferroni-corrected *post hoc t*-tests, *P* = 0.05/3). Then, to determine the brain regions with significantly altered nodal global and local efficiency, ANOVA was performed on the AUC of nodal efficiency with false-discovery rate (FDR) correction (*q* = 0.05).

We performed multiple linear regression analyses to investigate the relationships among log-transformed normalized WMH volume, functional efficiency metrics and cognition, adjusted for potential confounders (age, gender, and education).

Furthermore, we conducted mediation analyses to test the primary hypothesis of whether functional efficiency metrics mediated the relationships between WMH volumes and cognition, controlling for covariates (age, gender, and education). The primary estimates of interest were the degree of the changes in the direct path between WMH volumes and cognition, labeled *c* in the bi-variate models and *c’* in the full mediating models, and the indirect path from WMH volumes to cognition through functional efficiency metrics: the product of paths *a* and *b*. We computed the bias-corrected 95% confidence intervals (CI) for the size of the mediating effects with bootstrapping (*k* = 1000 samples). The mediating effect is said to be present if the 95% CI does not contain zero. Mediation analyses were conducted in PROCESS for the SPSS framework.

### Support Vector Machine

Support vector machine (SVM) analysis is extensively applied in disease classification. Generally, the SVM procedure involves three stages: feature selection, classifier training and prediction. SVM starts with feature selection as the basis for classification to form a high dimensional space. In this study, statistically significant features, like global and local efficiency, nodal global and local efficiency, between groups were selected for SVM. Then, SVM conducts the classifier training to construct a hyperplane that optimally separates the classes. Last, the classifier is used to predict the class label when a new sample is added into the classifier. In this study, SVM analysis was performed on the functional efficiency data of group differences using a toolkit named LIBSVM^5^. Due to the small sample size, we used the leave-one-out cross-validation test to evaluate the mean accuracy rate differentiating WMH-CIND from WMH-NC. The performance of a classifier can also be quantified using sensitivity, specificity and the area under the receiver operating characteristic curve (ROC) according to the results of cross-validation. Note that the specificity represents the proportion of WMH-NC subjects correctly predicted, while the sensitivity represents the proportion of WMH-CIND subjects correctly predicted.

## Results

### Demographic and Clinical Characteristics

Demographic and clinical variables for the HC, WMH-NC and WMH-CIND groups are summarized in Table 1. There were no significant differences in age, gender or years of education among the three groups. WMH, PWMH, and DWMH volumes in WMH-NC and WMH-CIND subjects were significantly higher than those in HCs (*P* < 0.001). The WMH-CIND group exhibited poorer performance on the MMSE (*P* < 0.001) and MoCA-BJ (*P* < 0.001) tests and showed worse episodic memory (*P* < 0.001), visuospatial function (*P* = 0.005), information processing speed (*P* < 0.001), language function (*P* < 0.001) and executive function (*P* < 0.001) than the HC and WMH-NC groups (details of cognitive performance in Table 1).

**TABLE 1:** Demographic and neuropsychological data.

**Items**	**HC (*n* = 38)**	**WMH**		***F/χ^2^/H***	***p***	***Post hoc analyses***		
		**NC (*n* = 36)**	**CIND (*n* = 38)**			**HC VS WMH-NC**	**HC VS. WMH-CIND**	**WMH-NC VS. WMH-CIND**
**Demographics**								
Age (years)	61.34 ± 1.16	65.03 ± 1.21	64.84 ± 1.27	2.950	0.056^b^	–	–	–
Education (years)	11.55 ± 0.36	11.25 ± 0.61	10.24 ± 0.52	1.902	0.154^b^	–	–	–
Gender (male/female)	20/18	15/21	23/15	2.650	0.266^a^	–	–	–
**Neuroimaging characteristics**								
Total brain volume (cm^3^)	1037.85(988.77, 1207.65)	1057.91(999.27, 1153.86)	1128.51(1045.41, 1234.87)	4.051	0.132^c^	–	–	–
WMH (mm^3^)	276.73(147.06, 572.46)	962.87(366.86, 2494.06)	2343.59(1109.30, 4729.15)	55.647	<0.001^c*^	<0.001^∗^	<0.001^∗^	0.376
PWMH	221.45(129.94, 468.37)	742.97(299.24, 1954.63)	1744.70(948.96, 3364.36)	53.866	<0.001^c*^	<0.001^∗^	<0.001^∗^	0.242
DWMH	30.93(17.22, 80.80)	133.89(29.50, 488.58)	315.80(108.48, 1251.60)	30.314	<0.001^c*^	<0.001^∗^	<0.001^∗^	0.999
**General cognition**								
MMSE	28.71 ± 0.21	28.61 ± 0.2	27.32 ± 0.39	7.768	<0.001b^∗^	0.804	0.001^∗^	0.002^∗^
MoCA-BJ	26.34 ± 0.25	25.78 ± 0.36	21.26 ± 0.57	45.440	<0.001^b*^	0.341	0.001^∗^	<0.001^∗^
**Composition *Z* scores of each cognitive domain**								
Episodic Memory	0.38 ± 0.10	0.04 ± 0.14	−0.42 ± 0.12	11.246	<0.001^b*^	0.048^∗^	<0.001^∗^	0.009^∗^
AVLT-DR	5.63 ± 0.33	5.19 ± 0.37	4.42 ± 0.34	3.188	0.045b^∗^	0.376	0.014^∗^	0.119
VR-DR (WMS)	9.58 ± 0.53	7.78 ± 0.58	5.71 ± 0.58	12.075	<0.001^b*^	0.026^∗^	<0.001^∗^	0.011^∗^
Visuospatial processing function	0.16 ± 0.07	0.20 ± 0.04	−0.36 ± 0.21	5.491	0.005^b*^	0.830	0.007^∗^	0.004^∗^
CDT	3.89 ± 0.06	3.97 ± 0.03	3.58 ± 0.12	6.858	0.002^b*^	0.495	0.006^∗^	0.001^∗^
VR-C	13.92 ± 0.06	13.86 ± 0.06	13.55 ± 0.23	2.002	0.140^b^	0.765	0.064	0.126
Information processing speed	0.37 ± 0.12	0.25 ± 0.12	−0.61 ± 0.12	20.250	<0.001^b*^	0.503	<0.001^∗^	<0.001^∗^
TMT-A (inverse)	0.025 ± 0.001	0.021 ± 0.001	0.016 ± 0.001	11.57	<0.001^b*^	0.049^∗^	<0.001^∗^	0.007
Stroop A (inverse)	0.064 ± 0.003	0.062 ± 0.002	0.046 ± 0.002	15.215	<0.001^b*^	0.693	<0.001^∗^	<0.001^∗^
Stroop B (inverse)	0.053 ± 0.002	0.055 ± 0.002	0.039 ± 0.002	15.734	<0.001^b*^	0.428	<0.001^∗^	<0.001^∗^
Language	0.28 ± 0.11	0.18 ± 0.10	−0.46 ± 0.14	11.718	<0.001^b*^	0.560	<0.001^∗^	<0.001^∗^
CVF	17.61 ± 0.67	17.17 ± 0.46	15.29 ± 0.69	3.971	0.022^b*^	0.620	0.009^∗^	0.036^∗^
BNT	51.42 ± 0.77	50.89 ± 0.91	45.79 ± 1.18	10.401	<0.001^b*^	0.700	<0.001^∗^	<0.001^∗^
Executive Function	0.30 ± 0.11	0.21 ± 0.11	−0.50 ± 0.10	16.614	<0.001^b*^	0.581	<0.001^∗^	<0.001^∗^
DST-backward	5.24 ± 0.20	4.92 ± 0.17	4.29 ± 0.24	5.476	0.005^b*^	0.280	0.002^∗^	0.036^∗^
TMT-B (inverse)	0.013 ± 0.001	0.012 ± 0.001	0.008 ± 0.001	13.722	<0.001^b*^	0.239	<0.001^∗^	<0.001^∗^
Stroop C (inverse)	0.035 ± 0.002	0.038 ± 0.002	0.027 ± 0.001	8.459	<0.001^b*^	0.280	0.004^∗^	<0.001^∗^

*Values are presented as the mean ± standard error (SE), median (interquartile ranges) or number (percentage). ^*a*^The *p*-value was obtained by χ^2^ test, ^*b*^the *p*-value was obtained by one-way ANOVA, and ^*c*^the *p*-value was obtained by Kruskal-Wallis one-way ANOVA. ^∗^ indicates a statistical difference between groups, *p* < 0.05. Abbreviations: HC, health control; NC, normal cognition; CIND, cognitive impairment without dementia; WMH, white matter hyperintensities. PWMH, periventricular-white matter hyperintensities; DWMH, deep-white matter hyperintensities; MMSE, mini mental state examination; MoCA-BJ, Beijing version of the montreal cognitive assessment; AVLT-DR, auditory verbal learning test-delayed recall; VR-DR, visual reproduction-delay recall; WMS, wechsler memory scale; CDT, clock drawing test; VR-C, visual reproduction-copy; CVF, category verbal fluency; BNT, boston naming test; DST, digit span test; TMT-A and TMT-B, trail making test-A and B; Stroop A, B, and C, Stroop Color and Word Tests A, B, and C.*

### Alterations in Network-Level Efficiency

**FIGURE 1:**
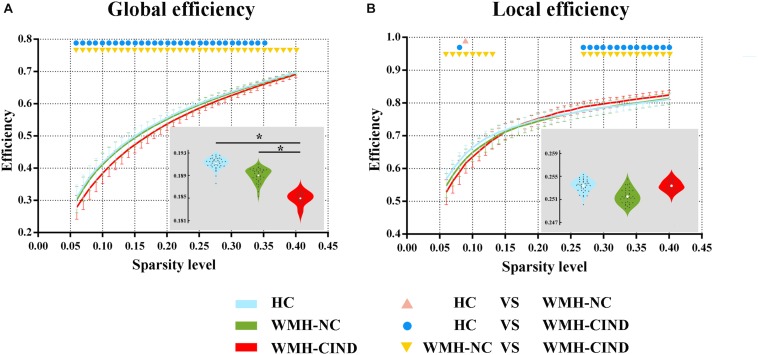
Global and local efficiency of the whole brain compared between HC, WMH-NC and WMH-CIND groups. **(A)** AUC of global efficiency in WMH-CIND subjects was significantly decreased compared with that of HCs and WMH-NC subjects (ANOVA, *P* < 0.001; Bonferroni-corrected, *P* < 0.05/3). **(B)** No significant differences in the AUC of local efficiency between the three groups were found (ANOVA, *P* = 0.471). Abbreviations: HC, healthy control; NC, normal cognition; CIND, cognitive impairment without dementia; WMH, white matter hyperintensities; AUC, area under the curve. ^∗^ indicates a statistical difference between groups.

First, we compared the global and local efficiency of the whole brain among the three groups. We found significantly decreased global efficiency in WMH-CIND subjects compared with HCs at sparsities of 0.06∼0.35 (*P* < 0.05, uncorrected) and WMH-NC subjects at sparsities of 0.06∼0.35 (*P* < 0.05, uncorrected), but no significant differences were found between HCs and WMH-NC subjects. Furthermore, the AUC of global efficiency in the WMH-CIND group was significantly lower than that in the HC and WMH-NC groups (ANOVA, *P* < 0.001; Bonferroni-corrected, *P* < 0.05/3) (Figure 1A). Although the local efficiency of the three groups in some sparsities was significantly altered, no significant differences in the AUC of local efficiency were found (ANOVA, *P* = 0.471) (Figure 1B).

**FIGURE 2:**
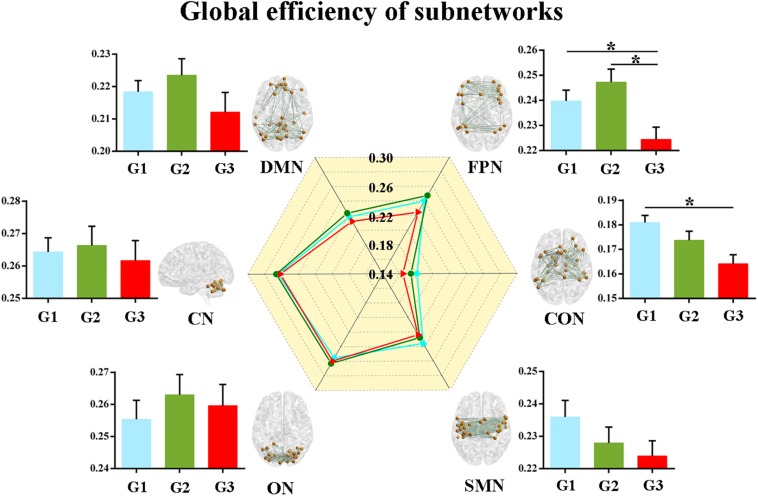
Global efficiency of the six subnetworks. WMH-CIND subjects showed lower global efficiency in the FPN than HCs and WMH-NC subjects (ANOVA, *P* = 0.004; Bonferroni-corrected, *P* < 0.05/3). The global efficiency of the CON in WMH-CIND subjects was significantly lower than that in HCs (ANOVA, *P* = 0.003; Bonferroni-corrected, *P* < 0.05/3). Abbreviations: HC, healthy control; NC, normal cognition; CIND, cognitive impairment without dementia; WMH, white matter hyperintensities; AUC, area under the curve; DMN, default mode network; FPN, fronto-parietal network; CON, cingulo-opercular network; SMN, sensorimotor network; ON, occipital network; CN, cerebellum network; G1 represents the HC group; G2 represents the WMH-NC group; and G3 represents the WMH-CIND group. ^∗^ indicates a statistical difference between groups.

**FIGURE 3:**
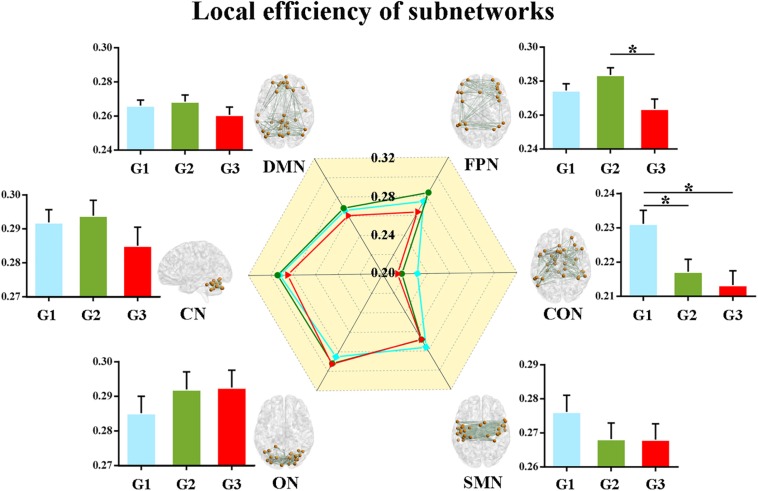
Local efficiency of the six subnetworks. Significantly decreased local efficiency in the FPN in WMH-CIND subjects compared with WMH-NC subjects was found (ANOVA, *P* = 0.014; Bonferroni-corrected, *P* < 0.05/3). HCs showed higher local efficiency in the CON than WMH-NC and WMH-CIND subjects (ANOVA, *P* = 0.004; Bonferroni-corrected, *P* < 0.05/3). Abbreviations: HC, healthy control; NC, normal cognition; CIND, cognitive impairment without dementia; WMH, white matter hyperintensities; AUC, area under the curve; DMN, default mode network; FPN, fronto-parietal network; CON, cingulo-opercular network; SMN, sensorimotor network; ON, occipital network; CN, cerebellum network; G1 represents the HC group; G2 represents the WMH-NC group; and G3 represents the WMH-CIND group. ^∗^ indicates a statistical difference between groups.

Second, we calculated the global and local efficiency of the six subnetworks. As displayed in Figure 2, WMH-CIND subjects showed lower global efficiency in the FPN than HCs and WMH-NC subjects (ANOVA, *P* = 0.004; Bonferroni-corrected, *P* < 0.05/3). In addition, the global efficiency of the CON in WMH-CIND subjects was significantly lower than that in HCs (ANOVA, *P* = 0.003; Bonferroni-corrected, *P* < 0.05/3). As shown in Figure 3, we found significantly decreased local efficiency in the FPN in WMH-CIND subjects compared with WMH-NC subjects (ANOVA, *P* = 0.014; Bonferroni-corrected, *P* < 0.05/3). Furthermore, HCs showed higher local efficiency in the CON than WMH-NC and WMH-CIND subjects (ANOVA, *P* = 0.004; Bonferroni-corrected, *P* < 0.05/3). No significant group differences in global and local efficiency were found in other subnetworks.

### Alterations in Node-Level Efficiency

**FIGURE 4:**
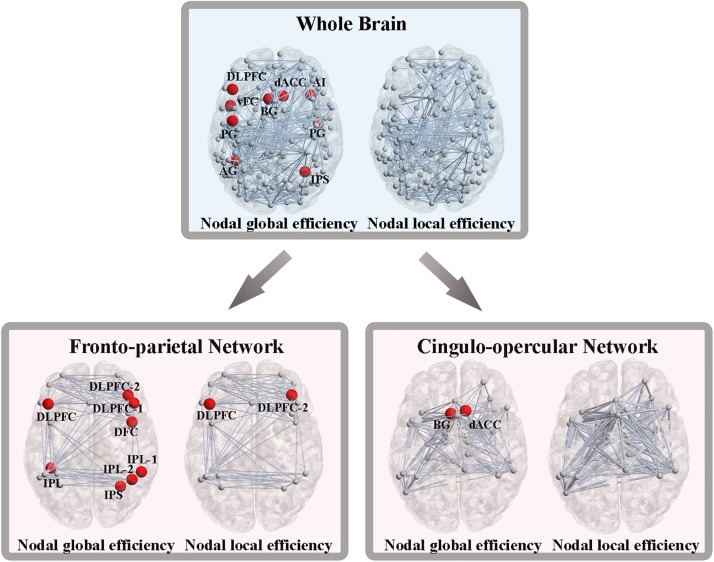
Altered nodal global and local efficiency. Abbreviations: DLPFC, dorsolateral prefrontal cortex; vFC, ventral frontal cortex; BG, basal ganglia; PG, precentral gyrus; AG, angular gyrus; dACC, dorsal anterior cingulate cortex; AI, anterior insular cortex; IPS, intraparietal sulcus; IPL, inferior parietal lobule; DFC, dorsal frontal cortex.

As we previously did for network-level efficiency, the AUC of nodal global and local efficiency were compared for each node. Out of the 160 ROIs, nine nodes showed significant group differences in nodal global efficiency, while no nodes were found to be significantly different in terms of nodal local efficiency (*P* < 0.05, FDR corrected). These nine nodes included the left dorsolateral prefrontal cortex (DLPFC), left ventral frontal cortex, left basal ganglia, left precentral gyrus, left angular gyrus, right dorsal anterior cingulate cortex, right anterior insular cortex, right precentral gyrus and right intraparietal sulcus (Figure 4).

From the perspective of the subnetworks, we have distinguished the significantly altered global and local efficiency of the FPN and CON. Next, at the node level of the FPN, eight nodes exhibited significant group differences in nodal global efficiency, and two nodes showed significant differences in nodal local efficiency (*P* < 0.05, FDR corrected) (Figure 4). At the node level of the CON, two nodes showed significant alterations in nodal global efficiency, while no nodes were found to be different in terms of nodal local efficiency (*P* < 0.05, FDR corrected) (Figure 4).

### Relationship Among WMH Volumes, Functional Efficiency Metrics, and Cognition

We performed multiple linear regression analyses to investigate the relationships among WMH volumes, functional efficiency metrics and cognition, adjusted for potential confounders (age, gender and education) in WMH-CIND subjects. First, we found that higher total WMH volumes and PWMH volumes were associated with lower scores for executive function (*β* = −0.398, *P* = 0.011; *β* = −0.353, *P* = 0.017, respectively) and information processing speed (*β* = −0.397, *P* = 0.017; *β* = −0.471, *P* = 0.002, respectively) (more details in Supplementary Table S1). Second, higher PWMH volumes were associated with lower nodal global efficiency in the left inferior parietal lobule (IPL) (*β* = −0.489, *P* = 0.006) and right dorsal frontal cortex (DFC) (*β* = −0.371, *P* = 0.039) (more details in Supplementary Table S2). Third, functional efficiency metrics were associated with cognitive scores for information processing speed (nodal global efficiency of the left DLPFC: *β* = 0.501, *P* = 0.003; nodal global efficiency of the left IPL in FPN: *β* = 0.419, *P* = 0.003; nodal global efficiency of the right DFC in FPN: *β* = 0.438, *P* = 0.004) (more details in Supplementary Table S3).

**FIGURE 5:**
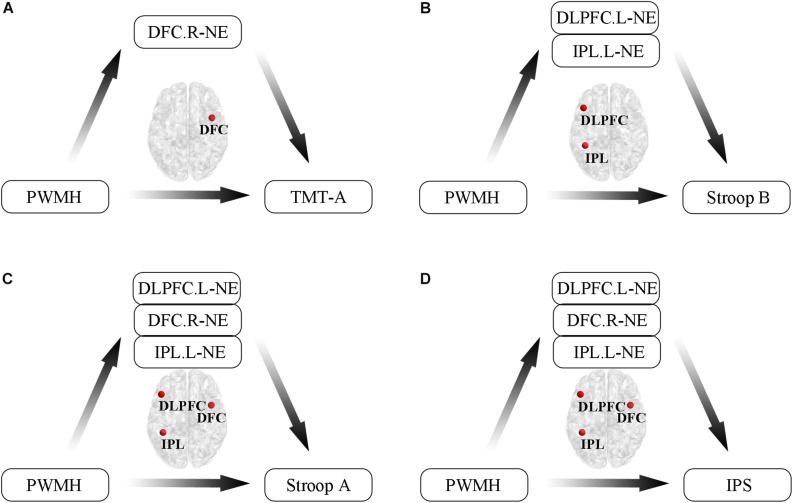
Mediation analyses to explore relationships between WMH volumes, functional efficiency metrics and cognition in WMH-CIND subjects. **(A)** Association between PWMH volume and TMT-A score was significantly mediated by nodal global efficiency in the right DFC in the FPN (indirect effect: –0.26; 95% CI: –0.74, –0.01). **(B)** Indirect effect of PWMH on Stroop B performance was significantly mediated by nodal global efficiency in the left IPL in the FPN and the left DLPFC (total indirect effect: –0.42; 95% CI: –1.12, –0.05). **(C,D)** The nodal global efficiency in the left IPL and the right DFC in the FPN and the left DLPFC mediated the association between PWMH volume and Stroop A performance (total indirect effect: –0.52; 95% CI: –1.15, –0.08) and information processing speed (total indirect effect: –0.36; 95% CI: –0.96, –0.003). Abbreviation: CIND, cognitive impairment without dementia; WMH, white matter hyperintensities; PWMH, periventricular-white matter hyperintensities; DLPFC.L, left dorsolateral prefrontal cortex; DFC.R, right dorsal frontal cortex; IPL.L, left inferior parietal lobule; NE, nodal global efficiency; TMT-A, trail making test-A; Stroop B, Stroop Color and Word Tests B; IPS, information processing speed.

Additionally, we conducted mediation analyses to explore whether functional efficiency metrics mediated the relationships between WMH volumes and cognition in WMH-CIND subjects, adjusting for age, gender and education. We found that the association between PWMH volume and the TMT-A score was significantly mediated by nodal global efficiency in the right DFC in the FPN (indirect effect: −0.26; 95% CI: −0.74, −0.01) (Figure 5A). A multiple mediator model demonstrated that the indirect effect of PWMH on the Stroop B test was significantly mediated by nodal global efficiency in the left IPL in the FPN and in the left DLPFC (total indirect effect: −0.42; 95% CI: −1.12, −0.05) (Figure 5B). The nodal global efficiency of the left IPL and right DFC in the FPN and the left DLPFC mediated the association between PWMH volume and Stroop A performance (total indirect effect: −0.52; 95% CI: −1.15, −0.08) and information processing speed (total indirect effect: −0.36; 95% CI: −0.96, −0.003) Figures 5C,D).

### Discriminative Analysis Based on Functional Efficiency Metrics

**FIGURE 6:**
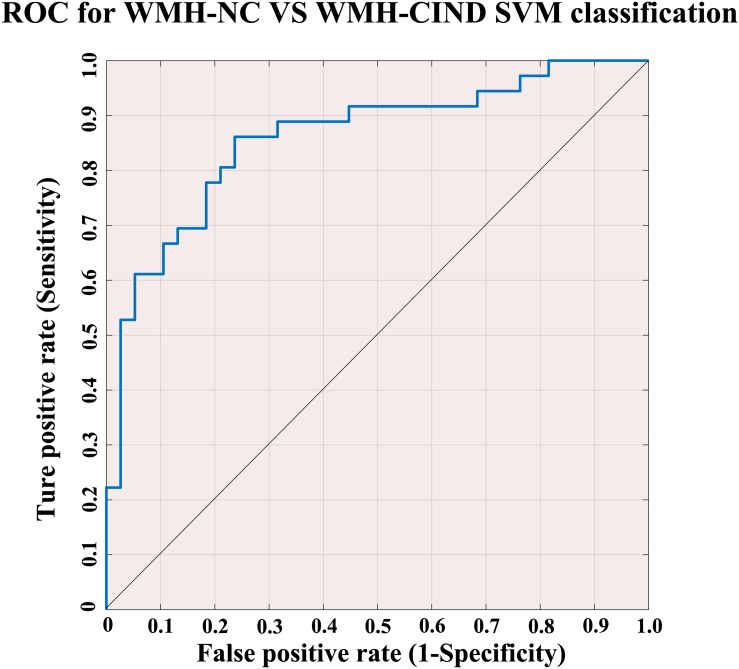
ROC for WMH-NC group vs. WMH-CIND group SVM classification. SVM analysis to classify the WMH-CIND subjects from subjects with only WMH with an accuracy of 81.08%, a sensitivity of 76.32% and a specificity of 86.11%. The area under the ROC curve was 0.86. Abbreviations: NC, normal cognition; CIND, cognitive impairment without dementia; WMH, white matter hyperintensities; SVM, support vector machine; ROC, receiver operating characteristic curve.

Using the classifier trained on differential functional efficiency metrics (Figures 1–4) and the leave-one-out cross-validation, SVM-based multivariate pattern analysis was able to classify the WMH-CIND subjects from subjects with only WMH with an accuracy of 81.08%, a sensitivity of 76.32% and a specificity of 86.11%. The ROC curve of the classifier was yielded, as shown in Figure 6. The area under the ROC curve was 0.86, indicating good classification power.

## Discussion

The present study first investigated topological patterns of resting state functional networks in WMH subjects with and without CI using a graph theory-based analysis method. The results showed that (i) WMH-CIND subjects displayed significant decreases in global efficiency at the levels of the whole brain, specific subnetworks (the FPN and CON) and certain nodes (mainly located in the FPN and CON), as well as decreased local efficiency in the abovementioned subnetworks and nodes; (ii) periventricular white matter hyperintensity (PWMH)-related impairment of information processing speed is mediated by a reduction in nodal global efficiency (brain regions involved: DLPFC. L, DFC. R, and IPL); and (iii) alterations in brain functional efficiency served as a useful neuroimaging marker for the identification of WMH-CIND subjects. Our findings added to the growing notion that alterations in large-scale brain functional networks might contribute to the onset of vascular CI.

### Significant Decreases in Connectivity Efficiency at Different Levels in WMH-CIND Subjects

As a direct topological measure of functional integration, global efficiency represents the capacity for parallel processing from distributed brain regions, while functional segregation refers to specialized processing within densely interconnected groups (the so called “modules”) and can be quantified by local efficiency (Rubinov and Sporns, 2010). As shown in Figures 1–4, the significant reduction in global efficiency in WMH-CIND subjects suggested potential damage to long association fibers that, in turn, impaired communication between crucial neural networks, especially those responsible for executive function and information processing speed (Ds, 2004; Sun et al., 2011; Schaefer et al., 2014). This damage finally gave rise to an impaired integration ability in WMH-CIND subjects’ brains. Similar results found in local efficiency reflect damage to neighborhood relationships between different nodes in the same modules, leading to a decrease in the level of specialized processing capacity. Specifically, involved brain regions were mainly located in the FPN and CON, and the alterations in both global efficiency and local efficiency revealed strong positive correlations with certain neuropsychological test scores (TMT-A, Stroop A/B and information processing speed tests). The FPN maintains and promotes homeostatic balance across multiple functional networks and serves as a flexible hub according to task demands (Cole et al., 2014). Previous findings showed that both the FPN and CON are related to information processing speed, executive function and attention (Niendam et al., 2012; Posner et al., 2016). The present findings confirmed the association between CI and functional alterations in large-scale brain networks in WMH subjects.

Interestingly, we found that there was no remarkable difference in the local efficiency between the WMH-CIND group, the WMH-NC group and the HC group at the whole brain level. A possible explanation is that some nodes may have intergroup differential local efficiency with statistical significance while others do not, which is confirmed by the following nodal efficiency analysis. Since global local efficiency is the average value of nodal local efficiency, a “neutralization reaction” may occur in the process of mean calculation. The more nodes involved, the more complicated the “neutralization” effect will be, eventually leading to an unpredictable or variable local efficiency value at the integral level. Moreover, compared to healthy subjects, WMH-NC subjects did not display altered functional connectivity patterns in our study, possibly suggesting a compensatory or maladaptive mechanism to maintain cognitive performance in those with mild pathological changes and symptoms (Prins and Scheltens, 2015). In addition, we did not observe the expected alterations in functional connectivity efficiency in the default mode network (DMN), which has been reported in several studies (Spreng et al., 2013; Dey et al., 2016). This inconsistency may result from different analytical methods. Voxel-wise FC analyses were performed in previous studies, while the present study focused on ROI-wise FC patterns.

### Mediation of the Association Between PWMH and Information Processing Speed Deficits by Nodal Global Efficiency

Previous studies have demonstrated that a disrupted topological organization of nodes is strongly correlated with CI or dementia, but the potential mechanisms are still unclear (Wen et al., 2005; Tuladhar et al., 2015; Yi et al., 2015). Here, we conduct a mediation analysis to further investigate the effect of nodal efficiency on the positive correlation between WMH burden and CI. We found that PWMH-related impairment of information processing speed is mediated by the reduction in nodal global efficiency, with corresponding brain regions (DLPFC. L, DFC. R, and IPL. L) involved (De Simoni et al., 2018). Similar studies elaborate on the relevance of information processing speed and injury to the above regions. DLPFC was considered an important control area to modulate the processing of social cognition and emotional cues (Weissman et al., 2008; Vanderhasselt et al., 2013), which can preserve cognitive tasks when emotional disturbances occur (Wessa et al., 2013). In addition, transcranial direct current stimulation to the left DLPFC can significantly increase impaired cognitive control in depression, which further illustrates its importance in information processing speed (Plewnia et al., 2015). The role of the IPL in meaning processing was confirmed by parametric manipulation (Chou et al., 2009), while the dorsal portion of the right inferior frontal cortex, especially the inferior frontal junction, is associated with specific attentional function, which is a major facet of information processing (Sebastian et al., 2016). Information processing speed, an important subdomain of human cognitive ability, is now commonly used as a behavioral indicator of the integrity of cognitive function and is specifically cited as a key component within the diagnostic framework for neurocognitive disorders in the DSM-5 (Li et al., 2010; Torrens-Burton et al., 2017). Reduced information processing speed is regarded as a highly robust feature of CI (Salthouse and Ferrer-Caja, 2003), and their relationship has been widely underpinned by numerous studies linking measured alterations in information processing speed to brain structure lesions (Wulfing et al., 2005; Jacobs et al., 2013; Hong et al., 2015). In addition, the structural network analysis also demonstrated that white matter microstructural damages were associated with the impaired information processing speed and the network global efficiency was shown to mediate the relationship between the white matter damage and cognitive dysfunction (Lawrence et al., 2014). From the functional network perspective, our study not only demonstrated that functional network efficiency played the “mediator” role, but also emphasized the importance of left dorsolateral prefrontal cortex, right dorsal frontal cortex, and left inferior parietal lobule in the mediation analysis.

Information processing speed is commonly impaired in subjects with WMH, and the mediation effects by nodal efficiency may describe the potential mechanisms underlying the link. The mediation effects of nodal efficiency were mainly found in frontal and parietal regions, the core nodes in the FPN. As PWMH burden increased, the nodal efficiency in the frontal and parietal regions decreased, suggesting that the FPN was disrupted. Then, the FPN could not integrate ongoing information across multiple functional networks very efficiently. Thus, information processing speed is commonly impaired in subjects with high PWMH burden.

Compared to previous studies, we set relatively tight inclusion criteria to eliminate the influence of irrelevant variables such as non-vascular factors, dementia and psychiatric symptoms as much as possible, ensuring, to a large extent, homogeneity across samples from the same group. Meanwhile, we also explored the internal relationships between diverse WMH burden levels and specific cognitive domains in a spatially oriented manner by applying the visual rating of WMH according to Scheltens et al. (Xu et al., 2019), which improves the relative accuracy of our study to a degree.

### Clustering Analysis Based on Support Vector Machine and Automatic Classification of WMH Patients

The early identification of WMH-CIND plays a vital role in the prevention of the onset and progression of CI and dementia. Several diagnostic approaches and potential detection methods have been developed (Van Maurik et al., 2017), including standard neuropsychological tests (Bai et al., 2010; Xu et al., 2010), neuroimaging markers (structural MRI markers, including those for hippocampal atrophy, ventricular volume and whole brain atrophy) and PET-CT markers (Nettiksimmons et al., 2010; Arbizu et al., 2018; Caminiti et al., 2018), electrophysiological tests (Imperatori et al., 2019) and biomarkers in cerebrospinal fluid (P-tau, tau, Aβ peptides, and Aβ42/P-tau ratio) (Van Harten et al., 2011; Llorens et al., 2016), with their respective strengths and weaknesses, as well as different prognostic accuracies and specificities. In recent years, resting-state fMRI (rs-fMRI) and diffusion MR markers have been increasingly used in the investigation of new markers of CI (Franzmeier et al., 2017). Based on our findings of intergroup FC differences, we evaluated their impact on the classification of WMH-CIND and WMH-NC by applying a machine learning algorithm called SVM analysis. Recently, Zhu et al. (2019) applied the altered whole-brain functional connections as the features to identify the WMH-CIND. From the “network property” perspective in our study, the differential functional efficiency metrics shows its unique value with an accuracy/sensitivity/specificity of 80.3%/ 76.5%/ 84.4%, respectively, highlighting the superior classificatory role of fMRI-SVM based automatic identification of WMH-CIND patients. To some extent, the topological properties may provide more information reflecting neural communication. I think the two approaches were complementary and both need to be applied in the future research. This strategy may have potential application value for clinical diagnosis.

## Limitations

Several limitations in this study need to be further addressed. First, the sample size was relatively small, which might lead to insufficient statistical power. Second, the topological organization of brain networks is affected by different parcellation strategies. The Dosenbach’s atlas used in this study was derived from a series of meta-analyses of task-related fMRI studies. Recently, two novel brain-wide graphs (264 ROIs reported in Power et al. (2011) and 333 cortical surface parcels reported in Gordon et al. (2016) may be used to further assess the suitability in the identification of WMH-CIND subjects. Third, a multicenter longitudinal study should be designed instead of simple cross-sectional research considering inevitable individual variation, and an individualized evaluation system for disease progression in WMH patients will ultimately be formulated in the future. Lastly, in this study, we only compared WMH with CIND and WMH without CIND, but didn’t involve CIND without WMH. We would add those subjects in the further studies.

## Conclusion

Our findings demonstrated that decreased global efficiency and local efficiency, especially in the FPN and CON, were prominently related to CI in WMH subjects, and decreased nodal global efficiency also mediated the association between WMH burden and CI. Discriminative analysis based on functional efficiency metrics further highlighted the superior classificatory role of large-scale functional networks in the identification of CI in WMH subjects. These findings provide novel insights into the altered large-scale brain functional networks of WMH subjects and may contribute to the investigation of markers of CI in the population.

## Data Availability Statement

The datasets generated for this study are available on request to the corresponding author.

## Ethics Statement

The studies involving human participants were reviewed and approved by the Ethics Committee of Nanjing Drum Tower Hospital. The patients/participants provided their written informed consent to participate in this study. Written informed consent was obtained from the individual(s) for the publication of any potentially identifiable images or data included in this article.

## Author Contributions

YX conceived and designed the experiments. HC, LH, DY, and QY performed the experiments. HC and LH analyzed the data. HC, LH, and YX drafted the manuscript. YX revised the manuscript. HC, LH, DY, QY, MG, RQ, CL, ML, LY, BZ, and YX collected the data and contributed the materials and analysis tools.

## Conflict of Interest

The authors declare that the research was conducted in the absence of any commercial or financial relationships that could be construed as a potential conflict of interest.

## Footnotes

1 https://sourceforge.net/projects/w2mhs

2 http://www.fil.ion.ucl.ac.uk/spm

3 http://www.nitrc.org/projects/gretna/

4 https://sites.google.com/site/bctnet/

5 http://www.csie.ntu.edu.tw/~cjlin/libsvm/
